# Treatment of ambulant patients by a general practitioner within a university hospital’s emergency department – follow-up study of patients’ behaviour shortly afterwards

**DOI:** 10.3205/000264

**Published:** 2018-07-04

**Authors:** Olaf Krause, Tanja Schleef, Maria Raker, Nils Schneider, Jutta Bleidorn

**Affiliations:** 1 Institute for General Practice, Hannover Medical School, Hannover, Germany

**Keywords:** emergency department, general practitioner, walk-in patients, primary care

## Abstract

**Aims:** Hospital emergency departments (ED) face an increasing number of outpatient cases. Therefore, Hannover Medical School employs general practitioners for the treatment of walk-in patients within the ED. Up to now, little has been reported on how these patients behave after treatment in the ED. This study aims to assess these patients’ behaviour after attending the ED, to find out how many patients consult a physician subsequently, and to explore patients’ utilisation of health care in case of similar health problems.

**Methods:** For this follow-up study, patients treated at Hannover Medical School’s ED during daytime (Monday – Friday) by a general practitioner (GP) within a period of six weeks in 2016 were subsequently followed up by phone interviews 10–15 days after their consultation. Main topics in the semi-structured interview guide were patients’ behaviour after leaving the ED, subsequent contacts with medical care, and how patients would behave in the future given similar symptoms. Data were transferred to a SPSS database, and descriptive data analyses were performed.

**Results:** In total, 171 patients were screened for inclusion, and 91 participated in the study. About half (n=48; 53%) of them were male, and the mean age was 46.6 years. After visiting the GP in the ED, 62 patients (68%) went directly home. Another 14 (15%) took up regular activities (e.g. work, university), while eight patients visited their usual GP practice. Within 10–15 days, 52 patients (57%) had had an appointment with a physician. In most cases this was a GP (n=34; 37%); 12 patients visited a specialist and six patients visited both a GP and a specialist. Physician appointments took place within an average of 3.4 days after treatment in the ED. In case of similar complaints, 37 patients (41%) would visit the ED again rather than visiting the GP, whereas 36% would consult the GP first, and 11% would visit a specialist first.

**Conclusion:** A noteworthy number of patients considered visiting the ED again with similar symptoms instead of visiting a GP in ambulatory care. Consequently our findings suggest that the ED itself plays a minor role in navigating patients’ utilisation of medical treatment.

## Zusammenfassung

**Zielsetzung:** Die Anzahl von ambulant zu behandelnden Patienten in den Notaufnahmen großer Krankenhäuser steigt stetig an. Aus diesem Grund setzt die Medizinische Hochschule Hannover Allgemeinärzte ein, um fußläufige Notfallpatienten in der Zentralen Notaufnahme (ZNA) zu versorgen. Bisher ist kaum bekannt, wie diese Patienten sich im Anschluss an den Besuch in der ZNA verhalten. Diese Studie hat zum Ziel, das Verhalten der Patienten nach der Konsultation in der Notaufnahme zu untersuchen, herauszufinden wie viele Patienten in der Folge einen Arzt aufsuchen, und das Vorgehen der Patienten beim Auftreten ähnlicher gesundheitlicher Probleme zu erfassen.

**Methode:** Für diese Follow-up Studie wurden Patienten, die in der Zentralen Notaufnahme (ZNA) der Medizinischen Hochschule Hannover während eines sechswöchigen Zeitraums tagsüber (montags – freitags) allgemeinmedizinisch versorgt wurden, 10–15 Tage nach Konsultation telefonisch nachbefragt. Wesentliche Punkte im semistrukturierten Interview waren das Verhalten der Patienten nach Verlassen der ZNA, weitere nachfolgende Arztkontakte, und das antizipierte Verhalten beim Auftreten ähnlicher Symptome. Die Daten wurden in eine SPSS-Datenbank transferiert und deskriptiv ausgewertet.

**Ergebnisse:** Insgesamt wurden 171 Patienten auf eine Teilnahme angesprochen, 91 Patienten nahmen teil. Etwa die Hälfte der Studienteilnehmer (n=48; 53%) war männlich, das mittlere Alter betrug 46,6 Jahre. Nach der allgemeinmedizinischen Konsultation in der ZNA gingen 62 Patienten (68%) direkt nach Hause. 14 (15%) Patienten nahmen direkt ihre regulären Aktivitäten wieder auf (Arbeit, Universität), während acht Patienten unmittelbar im Anschluss ihren Hausarzt aufsuchten. Innerhalb von 10–15 Tagen hatten 52 Patienten (57%) einen Arzt aufgesucht. Zumeist handelte es sich dabei um einen Hausarzt (n=34; 37%); 12 Patienten suchten einen Fachspezialisten auf, sechs Patienten suchten sowohl einen Hausarzt wie auch einen Fachspezialisten auf. Die ambulanten Arztbesuche erfolgten im Durchschnitt 3,4 Tage nach Besuch der ZNA. Beim Auftreten ähnlicher Beschwerden würden 37 Patienten (41%) eher als einen Hausarzt erneut die ZNA aufsuchen, während 36% als erstes einen Hausarzt und 11% als erstes einen Fachspezialisten aufsuchen würden.

**Schlussfolgerung:** Eine nennenswerte Anzahl der Patienten würde bei ähnlichen Beschwerden wieder die ZNA und nicht einen niedergelassenen Hausarzt aufzusuchen. In der Konsequenz deuten unsere Befunde darauf hin, dass die Notaufnahme selbst nur eine nachgeordnete Rolle in der Steuerung der Inanspruchnahme von medizinischen Leistungen durch Patienten spielt.

## Background

It is not just the perception of medical staff: statistics support that hospital emergency departments do in fact face an increasing number of outpatient cases. Studies have shown during the last few years that the number of these walk-in patients has risen in Germany [1], [2] as well as in other countries [3], [4], [5], despite the provision of outpatient care at various ambulatory levels. This has resulted in an ongoing public and highly emotional discussion in recent years about patients attending emergency departments (EDs) with so declared “minor health problems”. This is for example reflected in the poignant title of a study [6]: “nonurgent patients in emergency departments: rational or irresponsible consumers?” In that study, semi-structured interviews with 87 walk-in patients and 34 health professionals in several EDs were performed. The discrepancy of patients’ and health professionals’ perceptions was striking: Patients rationally focused on rapid appointments and access to technical features while health professionals e.g. looked out for the emergency of medical problems.

In theory, in the German health system a patient with a minor or less acute health problem (e.g. low back pain, gastroenteritis) should attend a general practitioner (GP). However, there is no mandatory gatekeeping by GPs in Germany. This means that patients have direct access to medical specialists if they so wish, and there is no barrier for patients with minor health problems to directly attend emergency departments even on weekdays when GP practices are open. Within the German health care system the role and responsibility of the EDs comprises immediately necessary diagnostics and treatment.

Numerous patients do declare themselves as “emergencies” and visit hospital EDs – even during GPs’ opening hours. A recent cross-sectional study (PiNo Nord) from 5 emergency departments in hospitals in Northern Germany questioned 1,175 walk-in patients about their reasons for attending the ED [7]. The reasons varied and included e.g. severe symptoms, unavailability of a general practice and the wish to get a maximum of diagnostic measures or specific treatments in a hospital ED. More than half of the respondents admitted that they did not think their health problem needed urgent treatment [7].

To cope with the growing number of walk-in patients, several community hospitals and so far two German university hospitals in Hamburg [8] and Hannover [9] employ GPs (i.e. specialist for general practice and general physicians) in their ED. Patients who consult the ED as “walk-in emergencies” are triaged and then treated by a GP with the aim to lead these patients back to ambulant care. At Hannover Medical School (MHH) ED, the GPs have treated about 1,500 patients during daytime (Monday to Friday, 10am to 6pm) in 2016, about 76% of the patients were self-referrals. The most common diagnoses were low back pain, gastroenteritis and hypertension. The majority (81%) of the patients were sent home, mostly with the advice to consult their GPs hereafter. Repeat patient visits were hardly found [9]. Thus, implementing GP care in the ED of a university hospital seems to be as a feasible opportunity to face the increasing number of walk-in patients [8], [9], [10].

Until now, it has not been investigated “how these patients behave” after receiving treatment by a GP in an ED of a university hospital and if they visit a GP afterwards. Thus, this follow-up study aimed:

- To assess what walk-in patients do after attending the ED (e.g. stay at home; visit other doctors)
- To find out how many patients consult a GP or a specialist after attending the ED
- To identify if these patients would attend the ED of a university hospital again for the same or similar health problems

## Methods

For this follow-up study, all outpatients who were treated at the Medical School’s ED by the general practitioners (GP) during daytime (Monday to Friday, 10am to 6pm) within a period of six weeks in July and August 2016 were invited to participate in the follow-up-study during their consultation. Exclusion criteria were: poor German language skills, cognitive impairment, not available for follow-up during study period and those with subsequent hospital admission. Participants provided written consent, and follow-up interviews were conducted by phone 10–15 days after their initial consultation.

For the interviews we used a self-developed semi-structured interview guide which was pretested with five patients to warrant understandability and feasibility. Main topics were patients’ behaviour after leaving the ED, subsequent contacts with medical care, and how patients would behave in the future with similar symptoms.

During the telephone interviews, data were recorded in written form on the questionnaire and transferred to a SPSS database. Reasons for the encounter and recommendations for subsequent outpatient consultations were taken from the discharge letter. Data analysis was performed descriptively. Free text answers were analysed descriptively by categorizing them based on similar answers given.

The study was approved by the Ethics Committee of the Medical School Hannover (Nr. 3024-2016). The study received no external funding and was financed by department’s resources.

## Results

Within the study period, 171 patients were allocated to GP treatment and screened for participation. In total, 91 patients agreed to participate, 80 patients declined or did not meet the inclusion criteria (non-participants, see Table 1 (Tab. 1)).

### Patients’ characteristics

About half (n=48; 53%) of participants were male, and the mean age was 46.6 years (range 19–89 yrs.). The majority of patients had a regular GP (n=85; 93%) in and around Hannover. Most participants (n=82; 90%) were self-referrals; they did not come due to the recommendation by an ambulant doctor. In contrast, nine patients (10%) were admitted, mostly by GPs and in one case by a specialist (for participants’ characteristics see Table 2 (Tab. 2)).

The majority (n=81; 88%) visited the ED as walk-in patients, whereas ten patients (12%) were admitted by ambulances. The most frequent complaints were musculoskeletal disorders.

Compared to all GP patients in the ED in 2016, study participants were more commonly male (53% in the follow-up study vs. 48% in all ED GP patients). No significant differences were found for age, sex and referral status.

### Recommendations made to patients

During GP consultation in the ED, most patients were recommended to visit a GP in the following days: 44 (48%) patients were recommended to visit their GP, 11 (12%) to visit a GP and a specialist, and 9 (10%) to visit a specialist. In total, 55 (60%) patients were advised to visit a GP (Table 3 (Tab. 3)).

### Patients’ behaviour following the ED visit

After GP consultation in the ED, 62 (68%) patients went home directly. Another 14 (15%) took up regular activities, i.e. went to school, university or work. Eight patients visited their usual GP practice directly afterwards, and four consulted other care services (Table 4 (Tab. 4)).

### Patients’ utilisation of health care providers following the ED visit

Within the time period from ED visit to the telephone interview (10–15 days), 52 (57%) patients made an appointment with a physician. In most cases this was a GP (34; 37%); 12 patients visited a specialist; six patients visited both GP and specialist, i.e. for internal medicine or orthopedics. Nine patients had made appointments for the next few days. Of the 55 of patients who were advised to visit a GP, 29 (53%) had realized a GP appointment by the time of their telephone interview, and four patients had only scheduled an appointment (Table 5 (Tab. 5)). Musculoskeletal disorders were associated with more doctor appointments: 16/24 (67%) of patients with musculoskeletal complaints visited a doctor within 10–14 days, compared to 57% in total.

**Figure 1:**
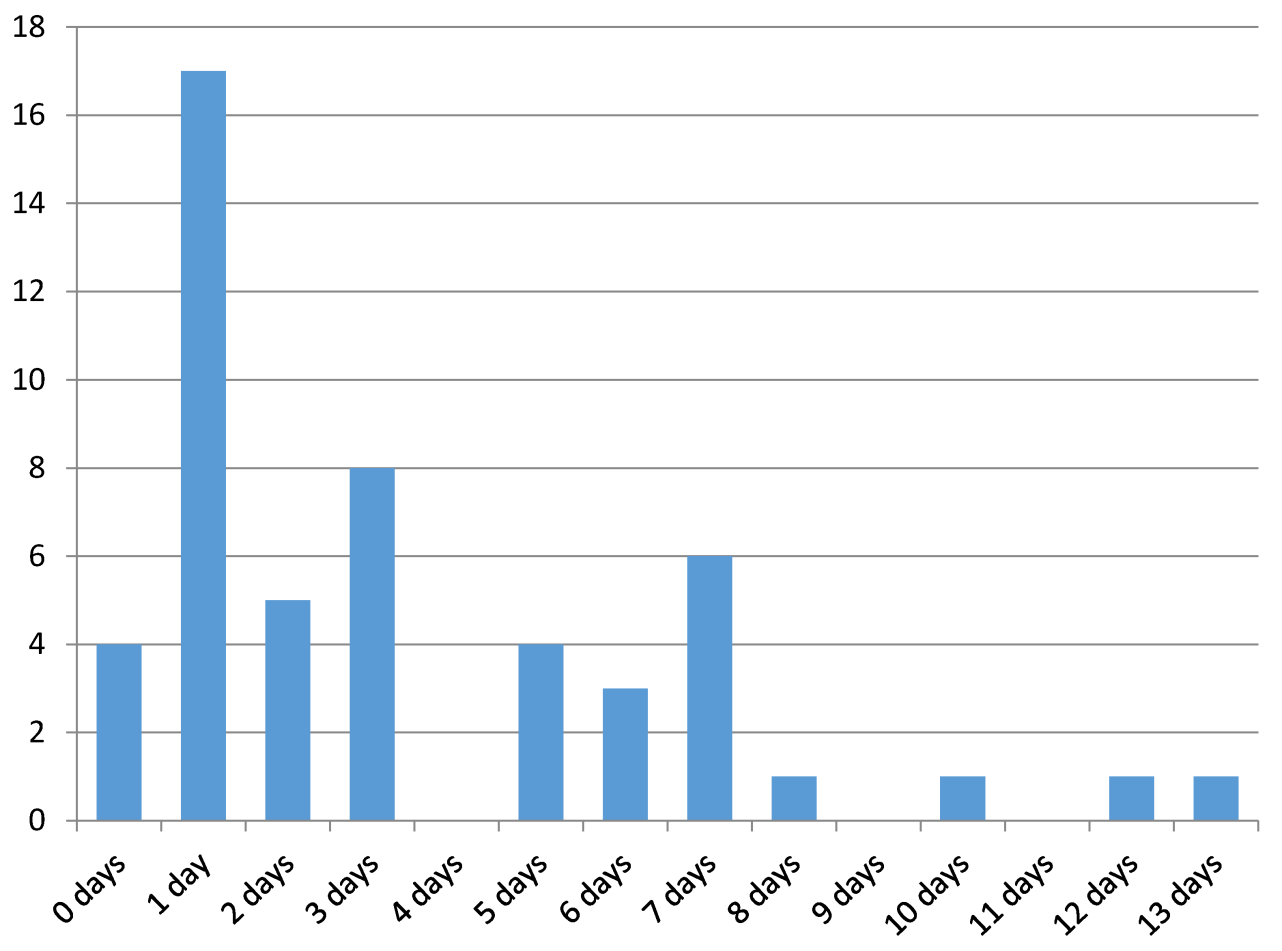
Time to doctor appointment after leaving the ED (av. 3.4 days)

Physician appointments after the initial outpatient treatment in the ED took place within 3.4 days on average. Male patients visited a doctor sooner than females (average 2.7 days for males vs. 4.4 days for females). In total, 34/52 (65%) had visited a doctor within the first three days after visiting the ED (Figure 1 (Fig. 1)).

### Patients’ behaviour in future situations

In case of similar complaints, 37 patients (41%) said they would visit the ED again rather than visiting the GP. 36% would consult the GP first, and 11% would visit a specialist at first.

As reasons for another ED visit, patients named the expectation of fast and comprehensive care, easy access outside of GP surgery opening times, and perceived low competence of GPs (Table 6 (Tab. 6)).

## Discussion

To our knowledge, this is the first study in Germany to assess the behavior of patients after receiving treatment by a GP within a university hospital ED. This study has focused on the two weeks after treatment in a university ED by a hospital employed GP: What have the patients done after visiting the ED? Have they gone back to a GP? Would they join the ED again in the case of the same symptoms?

Our results show that immediately after treatment most patients went back home and some patients even went to work or school/university, indicating that their complaints had not been too severe. Of the patients who were advised by the ED’s GP to contact their own ambulatory GP, half followed this advice and contacted their GP within two weeks. In the case of the same or similar symptoms, nearly half of the patients would attend the ED again.

An increasing number of patients with minor health problems expressed a preference for going straight to a hospital ED, so called “walk-in emergencies”, instead of consulting a GP’s practice. The reasons for the patients’ behaviour were investigated elsewhere, e.g. expecting better care and the possibility to be treated by specialists [11]. After attending the ED, patients consider their GP as their doctor in charge; about half of the study patients have seen their GP after the ED visit. Nevertheless, patients appeared to choose deliberately between the GP as part of the primary care system and the hospital ED, as most of our study patients would attend the university ED again in case of the same or similar symptoms. However, ambulatory GPs also appear to play a role, as they reportedly send their patients to the ED in case of uncertainty or to get a fast diagnostic measure from the ED, as recently reported in a qualitative study [12].

What are the consequences of our study? The aim to steer patients back to their GPs in primary care and away from the ED is a difficult target. Interventions have been tested in other countries to employ GPs in emergency departments, mostly in terms of GP post or cooperative GP offices [13], [14], [15]. Further measures that should be tested for walk-in patients are, e.g.: routine discharge letters for non-urgent patients to the GP to give better information; handouts for the patients to attend their GP first in case of minor symptoms; implementation of a complete general practice with full GP care directly next to the ED where walk-in patients can be separated from the other ED patients. Further and larger studies from university and non-university hospitals are needed to trace the patients’ paths before and after attending the ED, for understanding the patients’ reasoning behind their choices much better than we do now. Additionally, the placing of GPs in emergency departments [15] seems a smart way to deal with overcrowding in the ED due to walk-in emergencies, which can save scarce health care resources.

### Strengths and limitations of the study

Participants in this study did not differ from non-participants concerning age, sex and way of referral to the ED. Additionally, the participants seemed to be quite similar (age, sex, diagnoses) to patients in other ED studies [7]. Therefore, our study patients reflect the “typical” ED walk-in patients. In our study a semi-structured interviewer-assisted questionnaire has been conducted by phone, which we thought would yield a higher response rate than postal questionnaires. A final strength of this study was the prospective and patient centred design and implementation in a large university hospital ED.

However, some limitations must also be considered: The study took place in July and August 2016; during July there were school holidays in our region of Lower Saxony. This might have resulted in bias, yet the patient numbers analysed over the past years have shown a consistent occupancy rate in the ED of the MHH. Patients could only be registered for the study during the normal working hours of the employed GP at the ED of the MHH (10am to 6pm). Also, the phone interview may have led to reporting or social desirability bias, such as patients stating that they had made an appointment with the GP when this was not in fact the case. Another limitation is the limited time period. An extended period with more patients could have led to more significant results. Due to limited resources, this was not possible.

The study design did not include secondary data e.g. from the insurance system, for comparison to self-reported data. The transferability of the results to non-academic hospitals and other regions also remains unanswered. Finally, the patients’ GPs in ambulatory care have not been interviewed about their patients and their behaviour after attending the ED.

## Conclusions

The integration of GPs in an ED of a university hospital was implemented to cope with a growing number of walk-in emergencies. Up to a certain amount, this may lead to further appointments with the patients’ own GPs afterwards and subsequently bringing patients back into the primary care health system. Nevertheless, patients make their own decisions concerning their health and medical symptoms. Our results have shown that the majority of patients would attend the ED again rather than visiting a GP in ambulatory care. Thus, the ED itself seems to play a minor role in steering the patients’ utilisation of medical treatment. As a conclusion, the navigation of patients should be provided at a higher level to effectively control the access to emergency care (e.g. information campaigns, politics/policies, association of statutory health insurance). Another option would be accepting that many patients regard EDs as their first level support, and to respond to this demand by providing more resources within the ED and to adjust the framework conditions accordingly to the patients’ behaviour.

## Notes

### Competing interests

Olaf Krause and Jutta Bleidorn are part of the GP team to treat walk-in patients at the emergency department of Hannover Medical School. The other authors declare that they have no competing interests.

## Supplementary Material

Semi-structured interview guide (in German)

## Figures and Tables

**Table 1:**
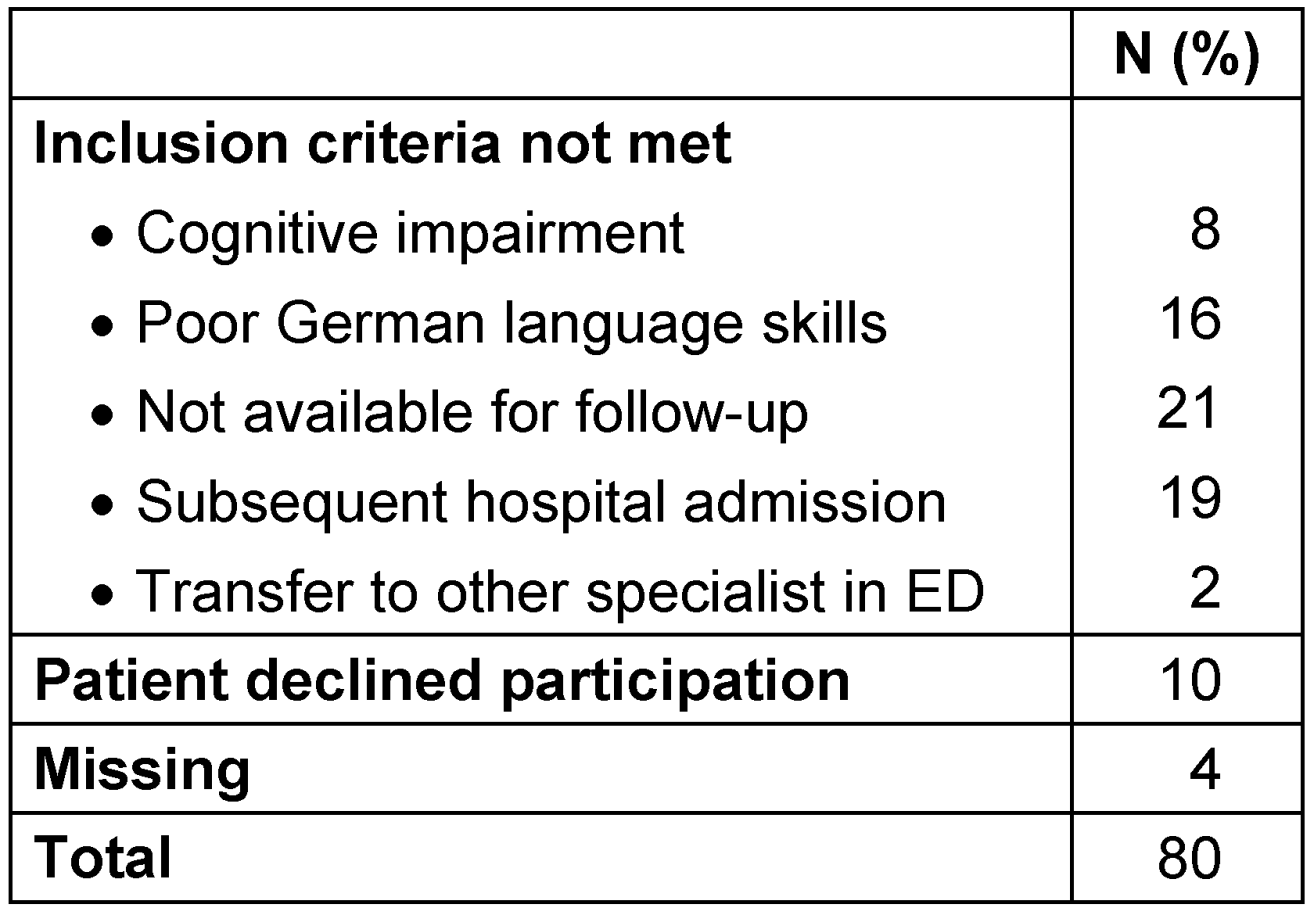
Non-participants

**Table 2:**
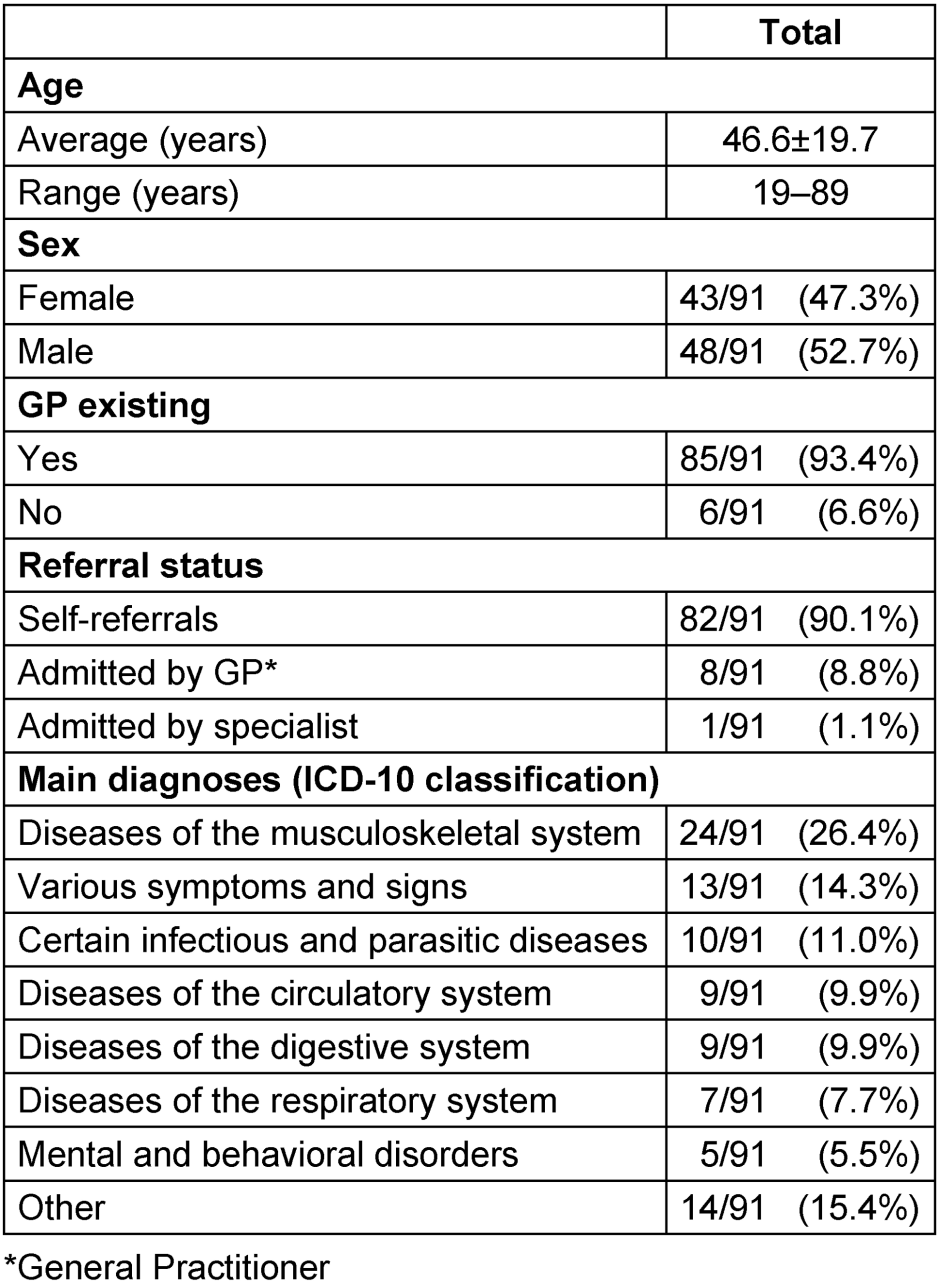
Patient characteristics (n=91)

**Table 3:**
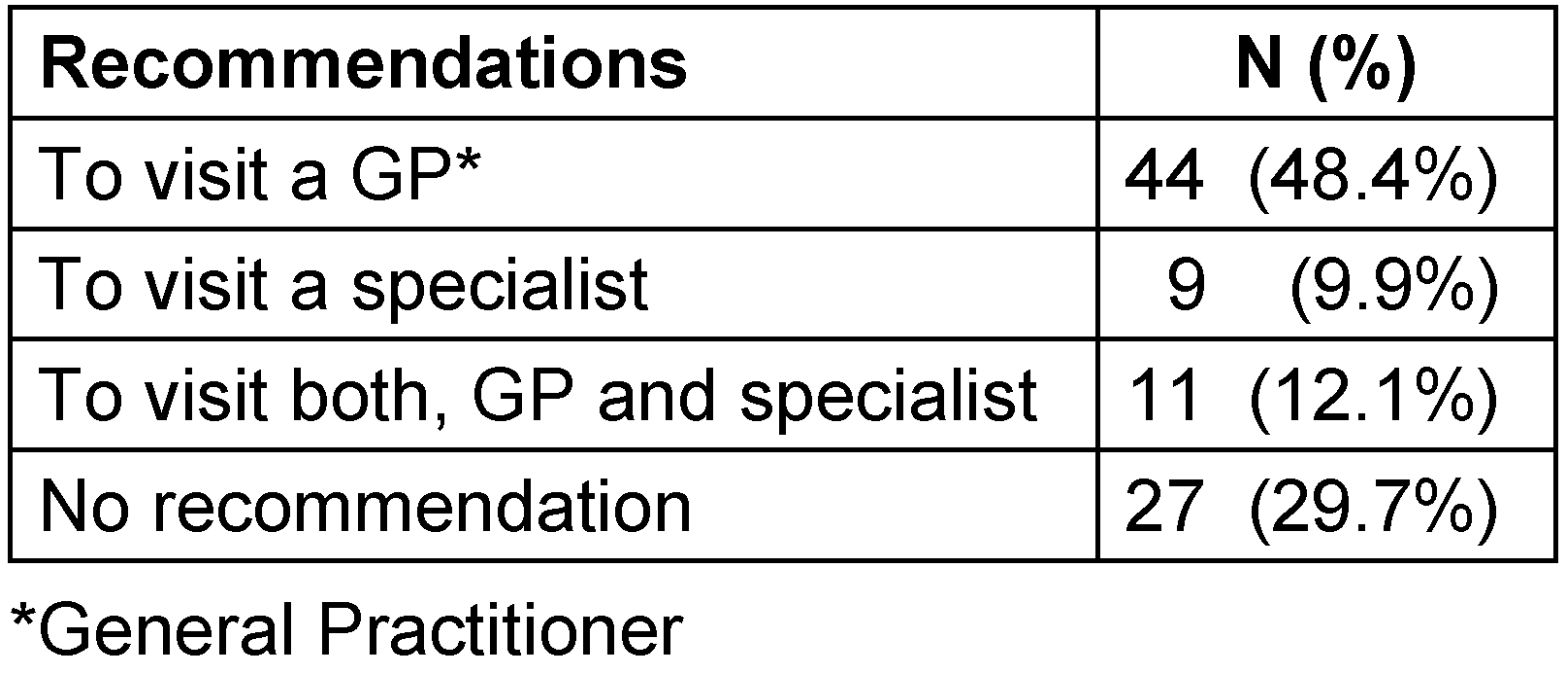
Recommendations for patients

**Table 4:**
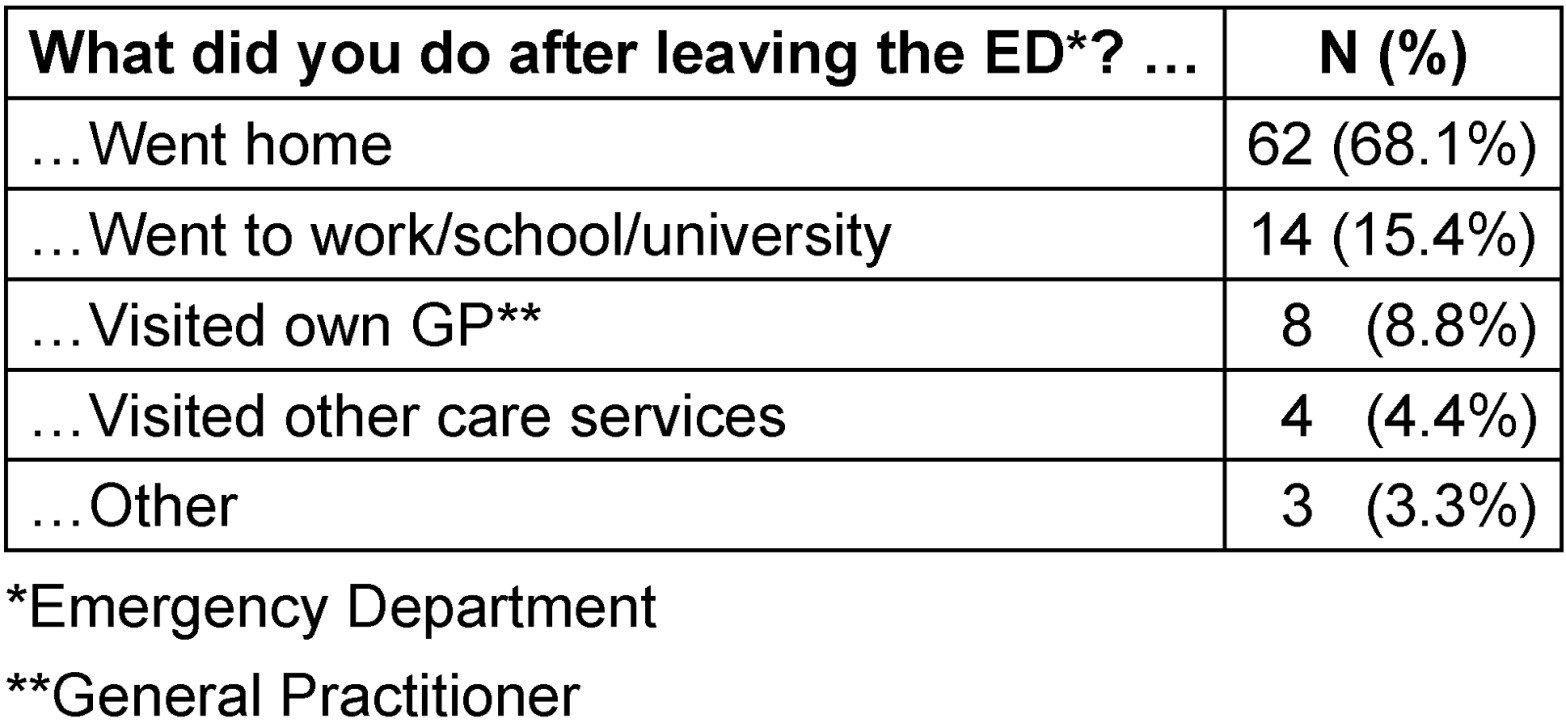
Patients’ performance after leaving ED

**Table 5:**
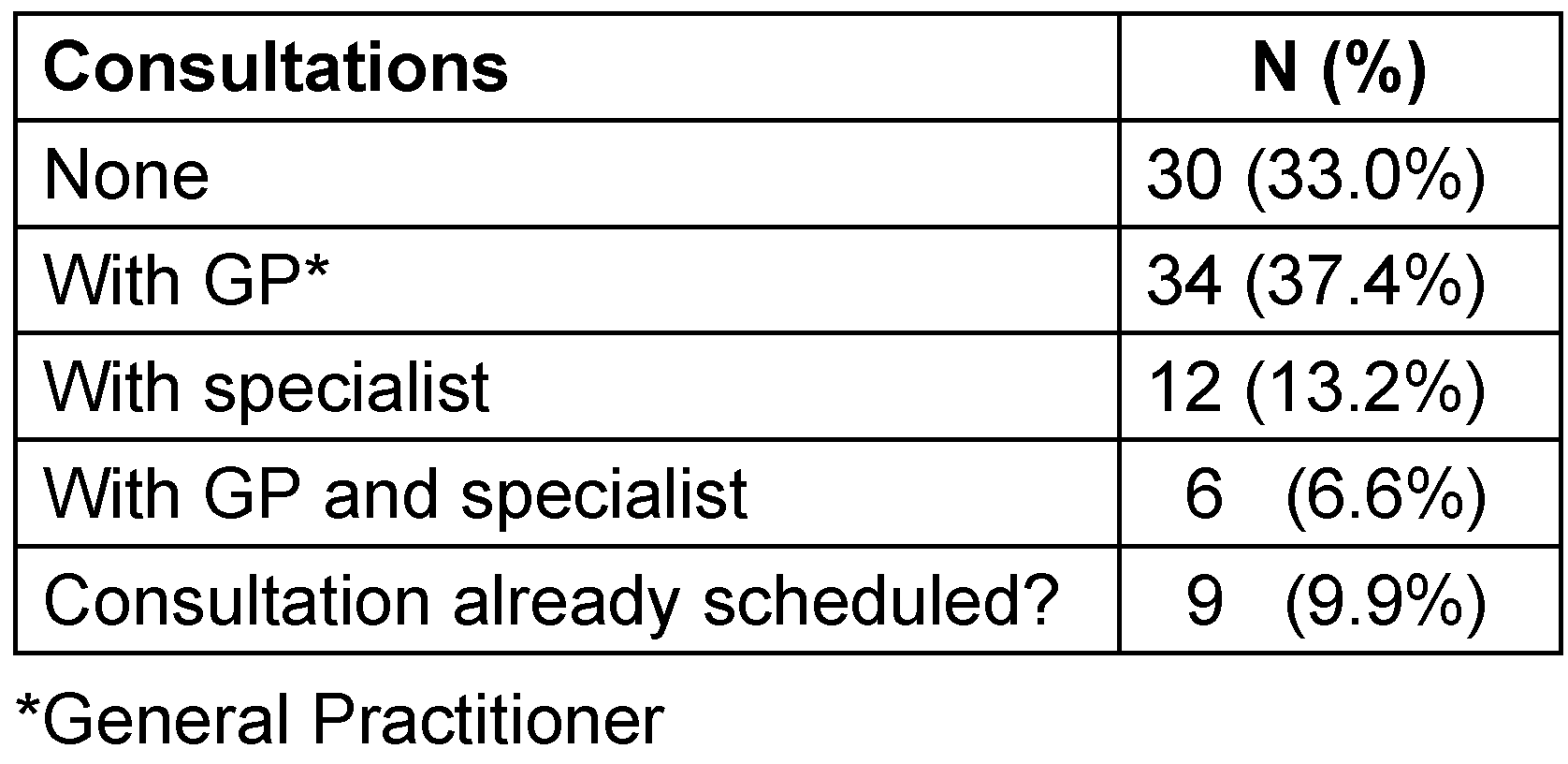
Physician consultations until telephone interview

**Table 6:**
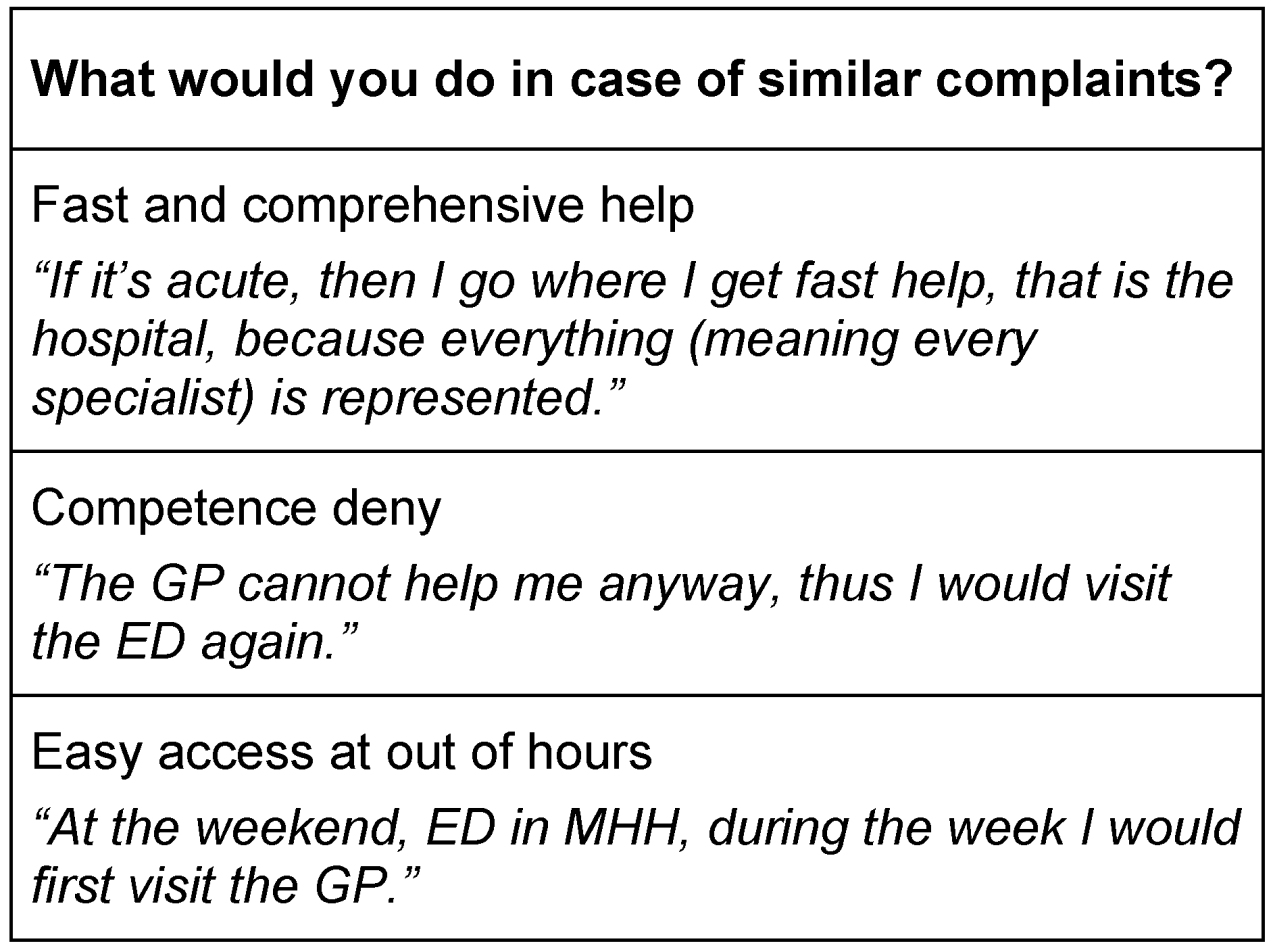
Reasons to visit ED again in case of similar complaints (exemplarily)
