# Supplementary material for: Treatment of ambulant patients by a general practitioner within a university hospital’s emergency department – follow-up study of patients’ behaviour shortly afterwards
Source: Ger Med Sci. 2018 Jul 4;16:Doc02. doi: 10.3205/000264 (PMC6044226; doi:10.3205/000264)
Supplement: Semi-structured interview guide (in German) [file GMS-16-02-s-001.pdf]

laufende Nummer: ______________

Patienten-ID: ______________

**Leitfaden zur telefonischen Befragung**
(10-14 Tage nach Besuch der allgemeinmedizinischen Notaufnahme)
**„Weiterversorgung von ambulanten, allgemeinmedizinisch behandelten Patienten nach dem Besuch einer universitären zentralen Notaufnahme“**

1. **Was haben Sie unternommen, nachdem Sie aus der Notaufnahme der MHH entlassen wurden?**
   ______________________________________________________________________________

2. **Wie haben sich die Beschwerden entwickelt, die zum Aufsuchen der ZNA geführt haben?“**
   (Bezug zum Arztbrief (Beschwerden))

   ☐ beschwerdefrei

   ☐ Verbesserung

   ☐ keine Besserung

   ☐ Verschlechterung

3. **Haben Sie nach Besuch der Notaufnahme einen Arzt aufgesucht?**

   ☐ ja ☐ nein

   wenn ja, welche Fachrichtung: Hausarzt ☐ Facharzt ☐
   Fachdisziplin:________________

   wenn nein: Arztbesuch beabsichtigt? Termin geplant?_________________________________

   **3. a Wie lange nach Ihrem Besuch in der Notaufnahme haben Sie einen Arzt aufgesucht?**

   Arzt aufgesucht am __________________ (nach x Tagen)

4. **Sie hatten ganz bestimmte Beschwerden, die Sie in die Notaufnahme geführt haben. Sollten ähnliche Beschwerden erneut bei Ihnen auftreten, wohin würden Sie das nächste Mal als erstes gehen?**

   ☐wieder in die Notaufnahme ☐zum Hausarzt ☐zum Fachspezialisten

   ☐zu keinem Arzt ☐sonstiges:_____________________________

   Gründe für erneuten Besuch der Notaufnahme:
   ________________________________________________________________________

   ________________________________________________________________________
